# Biophysical properties of the membrane influence spike initiation dynamics and neuronal excitability: a focus on Kv1 channels in myelinated axons

**DOI:** 10.1098/rspb.2025.0687

**Published:** 2025-07-16

**Authors:** Mohammad Amin Kamaleddin

**Affiliations:** ^1^ Temerty Centre for Artificial Intelligence Research and Education in Medicine, University of Toronto, Toronto, Ontario, Canada

**Keywords:** neuronal excitability, signal propagation, saltatory conduction, axon initial segment (AIS), spike initiation, Kv1 channels, action potential

## Abstract

Neurons and their subcellular compartments exhibit distinct forms of excitability. In 1948, Alan Hodgkin described different classes of neuronal excitability, each characterized by unique spiking responses to a constant stimulus. Despite these early insights, the mechanisms by which membrane properties influence spike initiation and excitability remain poorly understood. This review explores the nonlinear dynamics underlying spike initiation across excitability classes, emphasizing how these differences contribute to the neural encoding and processing of diverse information. Within a single neuron, compartments such as the soma, axon initial segment (AIS), and axon can exhibit functionally distinct excitability profiles due to differences in ion channel expression and membrane properties. For instance, the biophysical properties of myelinated axons, particularly the expression and distribution of voltage-gated potassium (Kv1) channels, play a key role in maintaining the directional fidelity of action potential propagation by facilitating orthodromic transmission and suppressing antidromic activity. These compartment-specific dynamics underscore the intricate design of neural systems to maintain the precision and efficiency of neural signalling. Moreover, perturbations in excitability are implicated in various neurological disorders, including epilepsy and chronic pain, highlighting the importance of maintaining physiological excitability profiles. By exploring these mechanisms, this review aims to provide insight into how alterations in membrane biophysics may inform future therapeutic strategies targeting excitability-related pathologies.

## 1. Introduction: a definition of neuronal excitability

The electrical properties of the membrane enable neurons to respond to stimuli. Excitability, in this context, refers to the ability and readiness of neurons to react to stimuli. Specifically, neuronal excitability is defined by the extent to which a given stimulus can alter the ionic balance across the membrane. Typically, this response manifests as spikes (action potentials) or as positive or negative shifts in membrane potential. For example, neurons that exhibit a stronger response to the same stimulus—evident in their higher rate of action potentials—are considered more excitable.

Spike generation represents an active neuronal response, and various aspects of spiking can be used to assess excitability. One approach is to calculate the number of spikes per unit of time, known as the spike rate or frequency, which can be measured by injecting depolarizing current. Alternatively, the voltage threshold for spike initiation can be measured to determine a neuron’s readiness to generate spikes. Broadly, neurons with more depolarized resting membrane potentials are closer to their voltage threshold, requiring a smaller stimulus for spike generation. Conversely, neurons with more hyperpolarized resting membrane potentials are generally less excitable, as they require stronger stimuli to initiate spikes.

Passive membrane properties, such as input resistance, also affect excitability. Lower input resistance necessitates a stronger current to drive the membrane potential to the threshold voltage. The value of the voltage threshold itself can be influenced by the density and activation properties of voltage-gated ion channels, such as voltage-gated sodium (Nav) channels. As discussed later, the spiking pattern in response to sustained depolarization conveys information about the interactions between different voltage-gated ion channels near the voltage threshold.

Spikes are typically initiated near the soma, at the axon initial segment (AIS), and then propagate orthodromically along the axon towards the axon terminals. However, under certain conditions, neurons can initiate spikes in distal regions of the axon. These ectopic spikes can backpropagate towards the soma. Ectopic spikes are frequently associated with pathological conditions such as epilepsy and chronic pain [1–5].

Nevertheless, ectopic spiking is not limited to pathological states. Studies have observed ectopic spiking in pyramidal neurons in the hippocampus and in both excitatory and inhibitory pyramidal neurons of the neocortex [6–10]. The role of ectopic spiking in neuronal computations under both physiological and pathological conditions remains an open area of investigation.

## 2. Different classes of neuronal excitability

**Figure 1.**
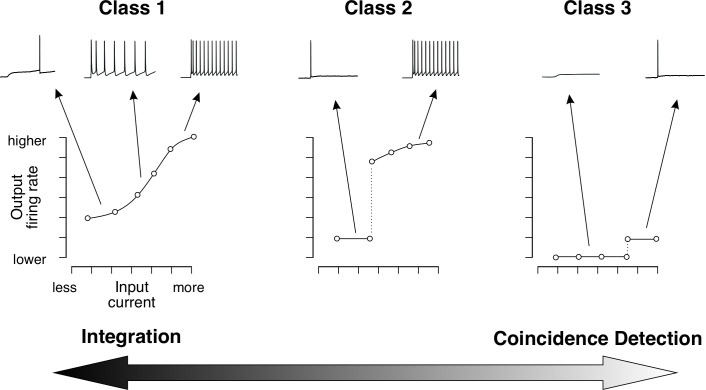
Different classes of excitability exhibit distinct firing behaviours. Spike initiation occurs when a fast-activating inward current depolarizes the cell, followed by hyperpolarization driven by a slower outward current. The net subthreshold current at steady state plays a crucial role in determining the class of excitability by influencing spike initiation dynamics. In class 1 neurons, the net subthreshold current at steady state is inward. The slow outward current does not significantly compete with the fast inward current, enabling repetitive spiking at very low rates. The input–output relationship is monotonic, meaning the firing rate gradually increases as stimulus strength grows. In class 2 neurons, the net subthreshold current at steady state is weakly outward. Here, the slow outward current competes with the fast inward current, preventing spiking at low frequencies. This results in a true threshold for repetitive firing, and intermediate response levels are not achievable. In class 3 neurons, the net subthreshold current at steady state is strongly outward. The inward current can overcome the slow outward current only during transient stimuli, such as abrupt depolarization. Consequently, class 3 neurons cannot fire repetitively. These differences in spike initiation dynamics enable neurons to serve distinct roles: integrators (class 1), coincidence detectors (class 3) or intermediaries along this functional spectrum. The plot illustrates the firing rate as a function of input stimulus strength. For further details, see [12].

In 1948, Alan Hodgkin injected sustained depolarizing currents into single axon fibres of the crab *Carcinus maenas* and identified three distinct classes of neuronal excitability based on spiking patterns [11]. To illustrate the differences in spiking patterns among these classes, input–output curves can be generated to show spiking responses to varying stimulus intensities (see figure 1).

Class 1 excitable neurons exhibit repetitive spiking, with a continuous spike frequency–stimulus intensity (f-I) curve. These neurons can spike repetitively even at low stimulus intensities. Class 2 excitable neurons also spike repetitively, but their f-I curve is discontinuous. Unlike class 1 neurons, class 2 neurons cannot sustain low-rate repetitive spiking. Class 3 excitable neurons do not spike repetitively, and as a result, the f-I curve is not defined for these neurons.

**Figure 2.**
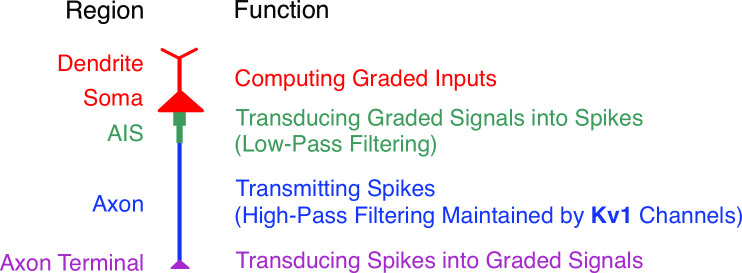
Distinct neuronal compartments exhibit specialized excitability to support their characteristic functions. Dendrites and soma receive graded inputs (excitatory and inhibitory postsynaptic potentials) from neighbouring synapses. The soma, along with the axon initial segment (AIS), integrates these graded signals and converts them into all-or-none spike-based signals. The axon transmits these spikes along its length and relays them to downstream neurons through synaptic transmission. Each region's specific function is supported by unique excitability properties, which are determined by the underlying biophysical and electrophysiological characteristics of that compartment.

The differences in excitability are not limited to comparisons between different neurons; distinct compartments within the same neuron can also exhibit different classes of excitability (figure 2). Intrinsic membrane properties, such as the differential expression of ion channels across compartments, influence spike initiation mechanisms and determine the class of excitability for each region. For instance, the activation of various ion channels, which may compete or cooperate with one another, regulates spike initiation. These nonlinear interactions, as will be discussed, depend on the specific characteristics of the stimulus.

## 3. Different classes of excitability distinguished by nonlinear dynamics of spike initiation

In 1948, Hodgkin characterized axons based on their distinct firing behaviours [11]. Fifty years later, Rinzel and Ermentrout developed mathematical models to explain how specific nonlinear dynamical mechanisms are associated with each firing behaviour and corresponding class of excitability [12–14].

From a biophysical perspective, differences in excitability class and spike initiation dynamics can be attributed to a balance—either competition or cooperation—between fast- and slow-activating currents near the voltage threshold [12]. In class 1 neurons, the net value of the slow-activating current is inward at subthreshold membrane potentials at steady state. This slow-activating inward current *cooperates* with the fast-activating inward current during spike generation, enabling repetitive spiking even at low stimulus intensities. In class 2 neurons, the net value of the slow-activating current is outward at subthreshold membrane potentials at steady-state, *competing* with the fast-activating inward current. However, once the stimulus is strong enough, the fast-activating inward current dominates, resulting in repetitive spiking. In class 3 neurons, similar to class 2, the slow-activating current is outward at subthreshold membrane potentials, *competing* with the fast-activating inward current. However, in class 3 neurons, the fast-activating inward current only dominates during transient changes in the stimulus, producing a brief spiking response at the onset of the stimulus. Sustained stimulation causes the slow-activating outward current to dominate, preventing repetitive spike generation.

Transitions between excitability classes can also be interpreted through changes in the balance between cooperating and competing currents. For example, a decrease in subthreshold inward current or an increase in subthreshold outward current can drive the transition from class 1 to class 2 or class 3 excitability [15]. Interestingly, such transitions are observed in axons as well. For instance, a decrease in subthreshold outward current mediated by voltage-gated potassium (Kv1) channels can shift axonal firing behaviour from transient to repetitive spiking, resulting in hyperexcitability [16–18].

## 4. Neural coding differences across classes of excitability and their role in information processing

The distinct spike initiation dynamics of different excitability classes translate into unique roles in neural coding. In class 1 excitable neurons, the cooperative nature of spike initiation dynamics enables them to function as low-pass filters [19]. These neurons are particularly sensitive to constant or slowly fluctuating stimuli (e.g. sustained pressure on the skin), as they integrate signals over time or space.

In contrast, the competitive spike initiation dynamics of class 2 and class 3 neurons allow them to act as high-pass filters [20]. Class 3 neurons exhibit the strongest responses to sudden or rapidly fluctuating stimuli (e.g. detecting edges of objects), making them adept at detecting temporal or spatial coincidence events in stimuli. This tuning specificity highlights the distinct coding strategies of each class: Class 1 neurons are tuned to the absolute magnitude of stimulus intensity. Class 3 neurons respond to the rate of changes in stimulus intensity. Class 2 neurons exhibit intermediate characteristics between these two extremes. Thus, class 1 and class 3 neurons represent opposite ends of a spectrum in information processing, functioning as integrators and coincidence detectors (or differentiators), respectively (figure 1).

Efficient information processing is vital for an organism’s survival and reproduction. Claude Shannon’s foundational work on information theory [21,22] provides a framework for understanding how the nervous system processes and encodes sensory information. This is particularly relevant for somatosensory processing, where sensory receptors must eliminate redundant information and compress a broad range of sensory inputs into their limited but dynamic physiological range [23]. By sampling only independent components of sensory information, the nervous system builds a factorial representation of the world, effectively mapping sensory inputs to neural representations. For this mapping to be efficient and information to be processed, preserving a proper neuronal excitability seems to be crucial [24].

Proper neuronal excitability plays a crucial role in this process. It is essential for sensory perception [25–27] and computations at subcellular regions [28,29]. However, maintaining proper excitability does not imply that spiking must always be regular. Irregular spiking is often required for neural coding to represent stimulus features effectively. Numerous studies have shown that neurons with physiological excitability can exhibit irregular spiking [30–32]. This irregularity can result from balanced excitatory and inhibitory synaptic inputs [33–35]. When excitatory inputs outweigh inhibitory ones, the neuron depolarizes, leading to regular spiking. Conversely, when inhibitory inputs counterbalance excitatory inputs over slower timescales, the membrane potential hovers near the voltage threshold. This dynamic allows the neuron to reach the threshold stochastically, akin to stochastic resonance [36], resulting in probabilistic and noise-driven irregular spiking.

## 5. Biophysical properties of neurons influence spike initiation dynamics

**Figure 3.**
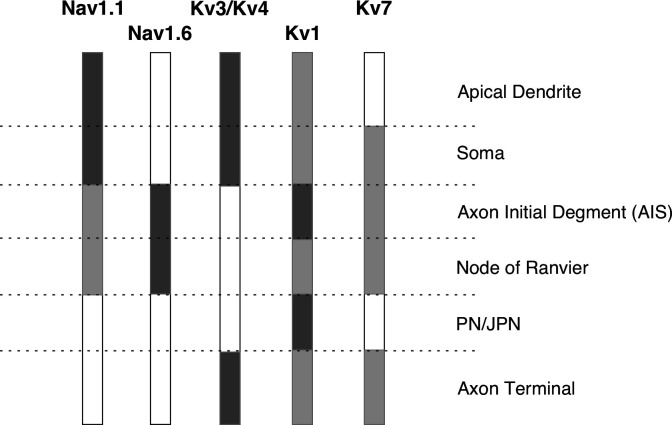
General distribution of subtypes of Nav and Kv channels in a typical pyramidal neuron of the mammalian cortex with myelinated axons. Different neuronal compartments express distinct combinations of ion channels, contributing to variations in excitability. Darker shades indicate higher expression levels. The numbers correspond to different ion channel subtypes. Note that this distribution may vary depending on the developmental stage and neuron type. For further details, see [38]. PN, paranode; JPN, juxtaparanode; Nav, voltage-gated sodium channel; Kv, voltage-gated potassium channel.

Morphologically distinct neuronal regions perform specialized functions. For example, dendrites primarily receive excitatory and inhibitory inputs from other neurons, and soma and dendrites cooperate to integrate these inputs [37]. The resulting graded changes in membrane potential are then transmitted to AIS, where action potentials (spikes) are typically initiated before propagating along the axon to the terminals. These specific functions are enabled by the differential expression of ion channels across neuronal compartments (figure 3).

Spike initiation can occur in various compartments of cortical neurons, with the dynamics and density of Nav channels playing a critical role in determining where initiation occurs. Spikes generally originate in the axon hillock and AIS due to the higher density of Nav channels in these regions [39–46] (for review, see [47]). This makes the axon hillock and AIS the most excitable regions of the neuron.

In addition to ion channel density, voltage-dependence of channel activation also influences the site of spike initiation. For instance, Nav channels in the AIS often exhibit lower activation voltages, further increasing excitability [41,48]. Notably, the density and dynamics of ion channels can adapt based on neuronal activity, a form of plasticity that affects their redistribution and activation properties. This plasticity, which can alter the subcellular localization of spike initiation, typically occurs over longer timescales [49,50]. Similar forms of plasticity in the AIS have been observed in its structural and functional properties, enabling homeostatic control of neuronal excitability (reviewed in [51]).

The structural characteristics of neuronal compartments (e.g. length, distance from the soma and surface area) also influence their computations and excitability. For example, neurons can initiate spikes even without a high density of functional Nav channels in the AIS, highlighting the importance of AIS structure [52]. The small size and low capacitance of the AIS, combined with its electrotonic isolation from the larger soma, facilitate easier charging and depolarization, enabling spike initiation. The length of the AIS and its distance from the soma also correlate with neuronal excitability. However, how the AIS’s relative position affects somatic excitability remains unresolved, with experimental and simulation studies suggesting varying outcomes [53,54]. Further research is needed to clarify this relationship.

The differential expression of ion channels at the AIS determines its biophysical properties (for review, see [51]). Among the various subtypes of Nav channels at the AIS, Nav1.6 is the most highly expressed subtype [55] and has the lowest activation threshold [41,56]. Consequently, Nav1.6 is the primary contributor to spike initiation in most neurons in the mammalian brain. In addition to its high density in this region, Nav1.6 is localized distally (farther from the soma) within the AIS [43,57] and this electrical segregation from the soma supports its role as the key ion channel in spike initiation [43,45,58,59].

In contrast, Nav1.1 and Nav1.2, which have higher activation thresholds [43], are located more proximally (closer to the soma) within the AIS [43,55,60]. These channels are primarily involved in facilitating the backpropagation of ectopic spikes from the axon to the soma and dendrites [43].

In addition to Nav channels, various subtypes of Kv channels are expressed at the axon initial segment (AIS) [55,61]. Kv1 channels (Kv1.1 and Kv1.2) and Kv7 channels (Kv7.2 and Kv7.3) are typically activated at voltages below the spike threshold, thereby suppressing spike initiation by competing with Nav channels [62,63] (for review, see [64]). Blocking Kv1 channels can alter spike initiation dynamics, promoting a transition from class 3 to class 2 or class 1 excitability [17,18]. Additionally, Kv1 channels play a role in shortening spike width [65,66] and are proposed to link intrinsic excitability with synaptic strength [67].

Kv7 channels are involved in setting the resting membrane potential, preventing slow depolarization that could inactivate Nav channels [68,69]. Kv2 channels (Kv2.1 and Kv2.2), on the other hand, are activated at voltages above the spike threshold and are preferentially activated by spikes, promoting the repolarization of action potentials [70]. Similarly, Kv3 channels, which are activated at suprathreshold membrane potentials, are expressed along the axon and at axon terminals [71]. Based on Hodgkin’s three classes of excitability, suprathreshold currents likely do not directly affect spike initiation. Therefore, Kv1 and Kv7 channels are more likely to influence spike initiation dynamics near the spike threshold.

Voltage-gated calcium (Cav) channels also play a role in the computations at the AIS by shaping neuronal firing patterns [51]. Cav2.3 channels are activated at subthreshold membrane potentials and modulate spike initiation by promoting after-depolarization [72]. Conversely, Cav2.1 and Cav2.2 channels, activated at suprathreshold membrane potentials, suppress spike initiation by facilitating action potential repolarization [73]. Another example is the T-type Cav3.2 channels, which are necessary for burst firing patterns at the AIS in auditory brainstem interneurons [74].

## 6. Different neuronal compartments receive different inputs, and their processing requires distinct spike initiation dynamics

Spike initiation dynamics vary across different neuronal regions, and these variations are essential for enabling each region to respond selectively to the types of inputs typically received by that region [75]. For instance, the soma is designed to receive graded signals, such as excitatory postsynaptic potentials (EPSPs) and inhibitory postsynaptic potentials (IPSPs), from the dendrites. As a result, the soma tends to fire repetitively in response to sustained depolarization. In contrast, the axon is adapted to respond to abrupt depolarization, typically originating from the soma/AIS. Therefore, the axon spikes transiently, even when stimulated with sustained depolarization [66]. Indeed, the spike initiation dynamics of each neuronal compartment determine whether and how the compartment will respond to a given input.

## 7. Different excitability classes of neuronal compartments impact their filtering properties

The unique biophysical properties of each neuronal compartment shape their characteristic electrophysiological and filtering properties. Understanding these filtering mechanisms is crucial for investigating how graded inputs, such as EPSPs and inhibitory postsynaptic potentials (IPSPs), are integrated in the dendrites and soma, and how these signals are transformed into spike-based outputs that can propagate with high fidelity along the axon.

In pyramidal neurons of the cortex, synaptic inputs are integrated nonlinearly [76]. The presence of voltage-gated ion channels in dendrites modulates inputs over both time and space. When inward currents are involved, this modulation amplifies depolarization, producing a response greater than the sum of individual synaptic inputs [76–79]. The passive cable filtering of EPSPs during dendritic propagation is influenced by synaptic timing and the interplay of synaptic depolarization with voltage-gated ion channels. For instance, Kv1 channels activated by dendritic EPSPs promote membrane repolarization in a voltage-dependent manner, reducing synaptic integration time and enhancing spike precision. This shift transforms the neuron’s operating mode toward coincidence detection [80] (figure 1). Remarkably, synaptic input integration on the same dendritic branch is nonlinear, whereas integration across different dendritic branches or in the soma tends to be linear [79,81–83].

EPSPs originating in the apical dendrites are prolonged as they propagate towards the soma [84]. However, the effects of cable filtering on somatic EPSP timing are offset by a high density of hyperpolarization-activated cyclic nucleotide-gated (HCN) channels in apical dendrites. These channels reduce temporal summation in the soma, ensuring precise signal processing in cortical pyramidal neurons [84].

The site of spike initiation also influences low-pass filtering along the axon. When spikes are initiated farther from the soma along the axon, the electrical isolation of the initiation site from somatodendritic currents improves [85]. Conversely, when the spike initiation site is closer to the soma and dendrites, the voltage threshold for spike initiation increases [86]. Additionally, Nav channel inactivation decreases progressively along the axon, which reduces temporal summation of synaptic potentials and enhances low-pass filtering [85].

## 8. Biophysical features of the axonal membrane impact its excitability and filtering properties

Understanding the filtering properties of the axon is essential for elucidating its role in neural signal transmission. One key aspect is the strong electrotonic coupling between the soma and axon [76]. This coupling allows passive propagation of large plateau potentials from the soma to the axon, which can inactivate Nav channels in the axon [87]. Similarly, the coupling may contribute to the failure of orthodromic spike propagation when spikes are initiated in the AIS [88–90]. In this context, electrotonic coupling can hinder efficient spike transmission along the axon [76].

Another important aspect of axonal membrane excitability in signal transmission is the modulation of spike-evoked neurotransmitter release by subthreshold changes in the presynaptic membrane potential. Both depolarization and hyperpolarization can facilitate synaptic release, but through distinct mechanisms and under different temporal dynamics. Depolarization-induced facilitation enhances synaptic strength over a relatively slow timescale by elevating baseline intracellular calcium levels and/or inactivating Kv1 channels [65,91]. In contrast, hyperpolarization-induced facilitation operates on a much faster timescale by relieving inactivation of Nav channels, thereby increasing the amplitude of the subsequent action potential in the axon [92].

Another work has extended this framework by demonstrating that axonal Nav channels can also function as detectors of input synchrony with millisecond precision [93]. Action potentials generated by highly synchronous inputs undergo less Nav channel inactivation, leading to larger axonal spike amplitudes and greater presynaptic calcium influx. This mechanism enables context-dependent modulation of synaptic transmission, allowing spikes driven by synchronous inputs to produce stronger postsynaptic responses compared with those driven by asynchronous inputs. These findings suggest that axonal sodium channels contribute not only to analogue-digital facilitation of synaptic strength, but also to the encoding and transmission of input synchrony across cortical networks.

In Purkinje cells, spike propagation failure during saltatory conduction is observed only at very high discharge frequencies [88,90]. Such propagation failures have been demonstrated across both invertebrates and vertebrates [94–98]. However, in pyramidal cells of the neocortex and hippocampus, spike propagation failure is much less common [99–101]. Another factor influencing axonal signal fidelity is the slow inactivation of Nav channels, which plays a critical role in ensuring reliable spike propagation along the axon. This slow inactivation depends on the neuronal firing characteristics, which vary across conditions and cell types [102].

Axons are capable of transmitting both suprathreshold spikes and subthreshold depolarizations, with the latter having significant implications for synaptic function. Evidence suggests that somatic subthreshold depolarizations can propagate along the axon for hundreds of micrometers. This is due to the large length constant of the axon—the distance over which voltage decays to approximately 37% of its original value [8,65,103–106]. However, other studies [67,107,108] have questioned the extent of this propagation. Importantly, subthreshold signals can modulate spike-triggered neurotransmitter release at axon terminals [103,105,109]. This modulation is influenced by the kinetics of depolarization and the rate of firing at synaptic terminals [76,110].

In myelinated axons, axonal excitability is closely tied to the conduction velocity of spikes. Conduction velocity is determined by the time required for nodes of Ranvier to regenerate action potentials and is influenced by the morphology and distribution of voltage-gated ion channels. For instance, thicker axons exhibit faster conduction velocities due to their reduced intracellular (axial) resistance, enabling greater excitability and higher firing frequencies [111]. Other morphological features—such as the length of nodes of Ranvier, internode length, myelin thickness and even membrane stretch—can also significantly impact conduction velocity and axonal excitability [112–114].

## 9. Case study: role of voltage-gated potassium channels in reliable spike transmission in myelinated axons

In myelinated axons, spikes are re-initiated at each node of Ranvier after their initiation at the soma and/or AIS. This process ensures the reliable propagation of neural signals over long distances. However, spike propagation fidelity can be influenced by activity-dependent changes in axonal membrane excitability and conduction velocity. These changes, in turn, modulate the timing of spikes travelling from proximal initiation sites to distal axon terminals [47,98,106,115–117]. Additionally, alterations in biophysical properties can lead to spike propagation failure or the initiation of antidromic spikes, which backpropagate to the soma [118].

Despite these challenges, under physiological conditions, axons transmit spikes with high fidelity, avoiding ectopic spike generation. This raises an intriguing question: how does the axon maintain its reliability despite its high concentration of Nav channels? The answer lies in axonal excitability, particularly the role of Kv1 channels.

Traditionally, axonal excitability has been studied using bleb recordings, where the end of the axon is cut, and changes in membrane potential are recorded in response to current injections see table 1). However, this method can alter the physiological behaviour of the axon. Recent advancements have used optogenetics to stimulate opsin-expressing axonal membranes, allowing researchers to record axon-originated spikes at the soma without invasive manipulation.

**Table 1.** Summary of selected studies investigating axonal excitability. Many of these studies rely on bleb recordings performed after cutting the axonal ends.

recording configuration	notable findings and comments	reference
recording from suction-induced blebs from muscle fibres	the bleb membrane is not fully representative of the normal surface membrane from which it arose	[119]
bleb recording from layer 5 unmyelinated pyramidal neurons (up to 303 μm from the soma)	hyperpolarizing the somatic membrane potential increases the amplitude of the axonal spike but does not affect the amplitude of the somatic spike; the calculated length constant of the axon is 417 µm	[103]
bleb recording from layer 5 pyramidal neurons (45–310 μm from the soma)	low-threshold, slowly inactivating K^+^ currents containing Kv1.2 alpha subunits play a key role in spike initiation dynamics in intracortical axons	[66]
bleb recording from layer 5 pyramidal neurons (50–440 μm from the soma)	main axons are unmyelinated for approximately 200 μm; spikes are typically initiated in a region approximately 35−50 μm from the soma, before the axons become myelinated	[120]
whole-cell recording from the axon (50 µm from the soma)	comparison of spikes initiated locally versus distally relative to the recording site	[121]
bleb recording from unmyelinated mossy fibre axons in CA3 (up to 97 μm from the soma)	spikes are initiated in the proximal axon at approximately 20−30 μm from the soma, presumably due to a higher Nav density in the axon	[122]
bleb recordings of axons of L5 pyramidal neurons of rat somatosensory cortex (up to 110 μm from the soma)	action potential generation requires a high sodium channel density in the axon initial segment	[45]
high-speed fluorescence Na^+^ imaging of subthreshold potentials and spike propagation along the axon of L5 pyramidal neurons of rat somatosensory cortex	little difference is observed in action potential-evoked Na^+^ influx between the axon and soma	[123]
bleb recording from unmyelinated axons of layer 5 pyramidal cortical neurons (25–720 μm from the hillock)	the spike waveform undergoes a dynamic change along the short region of the AIS, which is then maintained along the axon	[65]
bleb recording from layer 4/5 pyramidal neurons (20–45 μm from the soma)	long-time pulses may inactivate Nav in the axon	[124]
cell-attached and whole-cell bleb recording from inferior olive neuron axons (130 μm from the soma)	spikes are almost reliably propagated along the axon	[110]
a review on axon bleb recording	the resealed cut end of the axon may increase its length constant	[125]
bleb recording from CA1 pyramidal neurons (up to 705 μm from the soma)	the calculated length constant for the axon of CA1 pyramidal neurons is 712 μm	[104]
bleb recording from layer 5 pyramidal neurons (approx. 127 μm from the soma)	GABAA receptors are expressed in axons, suggesting that GABA hyperpolarizes the axonal membrane potential	[126]
bleb recording from layer 4/5 pyramidal neurons (up to 358 μm from the soma)	long-time depolarization primarily induces spikes in the soma, whereas short-time pulses induce spikes in the axon	[75]
bleb recording from parvalbumin and somatostatin expressing interneurons in layers 2−5 (approx. 112 μm from the soma)	the expression of different subtypes of Nav channels is heterogeneous across a population of interneurons and neuronal regions	[127]
bleb recording from layer 4/5 pyramidal neurons (distance not specified)	both spikes and subthreshold potentials propagate between the soma and axon with high fidelity; in axonal blebs, values reported for resting membrane potential and spike dynamics are close to normal, suggesting normal functional properties	[128]
bleb recording from layer 5 pyramidal neurons (approx. 300 μm from the soma)	bleb recording is only possible at proximal regions of the axon where it is unmyelinated; neuromodulation may alter spike dynamics in the axon, affecting analogue-to-digital information processing	[129]
bleb recording from layer 6 pyramidal neurons (approx. 100 μm from the soma)	somatic and axonal sodium currents are primarily mediated through Nav1.2 and Nav1.6, respectively	[130]
bleb recording from cortical pyramidal neurons (approx. 350 μm from the soma)	somatic subthreshold potentials propagate into the axon over considerable distances (>350 μm); however, the spread of axonal subthreshold potentials into the soma is inefficient	[8]
using optogenetics to photostimulate ChR2-expressing axons of CA1 pyramidal neurons and recording antidromic spikes in the soma with a patch pipette	Kv1 channels in the axon act as high-pass filters, enabling reliable propagation of orthodromic spikes without initiating antidromic spikes	[16]

A study by Kamaleddin *et al.* [16] demonstrated that Kv1 channels are essential for maintaining class 3 excitability in axons. Using optogenetic techniques, the study showed that Kv1 channels act as a high-pass filter. During sustained, gradual depolarization, the slow-activating outward currents mediated by Kv1 channels counteract fast-activating inward currents, thereby suppressing spike initiation. Conversely, transient and abrupt depolarization activates fast inward currents, initiating spikes before the outward currents can respond.

Blocking Kv1 channels in distal axons induces antidromic propagation of axonal spike bursts towards the soma [131]. These findings underscore the importance of Kv1 channels in enabling axons to transmit spikes reliably without generating ectopic activity. By tailoring their response to stimulus kinetics, axonal Kv1 channels ensure that spikes are initiated only under appropriate conditions.

This case study highlights how the distinct ionic channel composition of the axonal membrane enables dynamic modulation of excitability, supporting the axon’s role in reliable and efficient signal transmission.

## 10. Conclusions and future directions

(1) The AIS and axon are integral components of neuronal signal processing, with unique biophysical and electrophysiological properties that influence neuronal excitability, spike initiation and signal propagation. The differential expression and distribution of ion channels across neuronal compartments underlie their specialized roles in integrating and transmitting electrical signals.
(2) In the AIS, Nav1.6 channels play a critical role in lowering the activation threshold for spike initiation, while other Nav channel subtypes, such as Nav1.1 and Nav1.2, facilitate backpropagation of spikes. Kv and Cav channels further modulate spike dynamics and excitability, with Kv1 and Kv7 channels being particularly important for maintaining spike fidelity near threshold potentials.
(3) The axonal membrane, with its distinct ionic channel composition and electrical coupling with the soma, governs not only spike propagation but also subthreshold signal transmission. These features enable the axon to dynamically adjust its excitability and filtering properties, ensuring reliable signal transmission over long distances. Kv1 channels, in particular, serve as a high-pass filter to prevent ectopic spike generation, highlighting their critical role in axonal signal fidelity.
(4) Neuronal compartments also demonstrate unique excitability classes, which shape their filtering properties and responses to inputs. Nonlinear integration of inputs in dendrites contrasts with the largely linear integration at the soma, facilitating diverse computational processes. Additionally, the interplay of passive and active filtering mechanisms further modulates signal propagation and spike timing.
(5) Overall, the distinct biophysical properties of the AIS and axon allow neurons to process and transmit information with high precision and reliability. These mechanisms are crucial for maintaining the functional integrity of neural circuits and enabling the complex computations necessary for behaviour and cognition. Understanding these processes at a deeper level not only sheds light on fundamental neuroscience but also provides insights into potential targets for therapeutic intervention in neurological disorders.

## Data Availability

This article has no additional data.
